# Interferon-α for Immune Modulation in Chronic Hepatitis B Toward Functional Cure

**DOI:** 10.3390/v17101358

**Published:** 2025-10-10

**Authors:** Asha Ashuo, Jia Liu, Zhenghong Yuan, Jieliang Chen

**Affiliations:** 1 Key Laboratory of Medical Molecular Virology (MOE/NHC), Research Unit of Cure of Chronic Hepatitis B Virus Infection (CAMS), Shanghai Frontiers Science Center of Pathogenic Microbes and Infection, School of Basic Medical Sciences, Shanghai Medical College Fudan University, Shanghai, 200032, China; 2 Department of Infectious Diseases, Union Hospital, Tongji Medical College, Huazhong University of Science and Technology, Wuhan 430074, China

**Keywords:** chronic hepatitis B, PEG-IFNα, immune modulation, combination therapy, HBV cure

## Abstract

Chronic hepatitis B (CHB) remains a major global health challenge, largely due to the persistence of covalently closed circular DNA (cccDNA) and impaired host immunity. Interferon-α (IFN-α), a key antiviral cytokine, not only directly restricts HBV replication but also orchestrates innate and adaptive immune responses. This review summarizes current advances in IFN-α-mediated immune regulation, highlighting its effects across diverse immune cell populations. Evidence indicates that IFN-α can reprogram immune responses to promote viral clearance, although clinical efficacy is limited by modest response rates and adverse effects. Recent progress in cytokine engineering, subtype research, and rational combination strategies—including nucleo(s/t)ide analogs, RNA interference therapeutics, antisense oligonucleotides, therapeutic vaccines, and beyond—has expanded opportunities to improve treatment outcomes. While challenges remain, these advances lay the foundation for optimizing IFN-α–based interventions and highlight IFN-α as a key driver for innovative therapies aimed at achieving a functional cure of chronic hepatitis B.

Hepatitis B virus (HBV) infection broadly threatens human health and life, and has always been a serious global public health issue that cannot be ignored. According to the World Health Organization (WHO), approximately 254 million individuals were living with chronic HBV in 2022, and an estimated 1.1 million people died from chronic hepatitis B (CHB) and HBV-related liver diseases, such as cirrhosis and hepatocellular carcinoma (HCC) [1,2].

Nucleo(s/t)ide analogs (NAs) and interferon-alpha (IFN-α) are currently the main drugs for the clinical treatment of chronic hepatitis B. NAs can effectively control HBV replication, but it requires long-term medication and discontinuation is prone to lead to relapse. Interferon (IFN), a class of natural cytokines with direct antiviral effects and immunoregulatory functions, was first discovered and named by Alick Isaacs and others in 1957. IFNs are mainly divided into types I, II, and III depending on their receptors. The IFN-I family includes multiple members, with interferon-alpha (IFN-α) and interferon-beta (IFN-β) being the most studied. IFN-α exerts its antiviral effects by specifically binding to the type I interferon receptors IFNAR1 and IFNAR2, initiating the transduction of downstream signaling pathways and further inducing antiviral effectors [3]. Meanwhile, IFN-α can regulate innate and adaptive immune cells in direct or indirect ways, playing a key role in the host antiviral immune response [4]. Currently, IFN-α, primarily the IFN-α2a and IFN-α2b subtypes, remains a valuable treatment option for a select cohort of chronic hepatitis B patients, which is typically characterized by a finite treatment duration and the potential to induce a sustained functional cure [5], defined as sustained undetectable HBsAg and HBV DNA with or without the development of anti-HBs after a finite course of treatment, and potential long-term benefits in reducing hepatocellular carcinoma incidence [6]. However, there are still certain problems such as significant side effects and limitations in the overall response rate among certain populations.

The establishment and clinical outcome of HBV infection are tightly linked to the host immune response. Here, we mainly summarize the current understanding of the immunoregulatory activity of IFN-α, including how it regulates and affects the functional activity of different immune cells, as well as recent developments of new strategies aimed at enhancing its efficacy in achieving functional cure.

## 1. Role of the Immune System and IFN in Controlling HBV Infection

The interferon (IFN) system is a cornerstone of antiviral immunity, orchestrating host responses to limit viral replication and spread. The IFN family comprises three types: type I (IFN-I), type II (IFN-II), and type III (IFN-III), with type I interferons playing a pivotal roles in antiviral defense. IFN-α, a key member of the IFN-I family, exerts its effects by binding to the IFNAR receptor, activating downstream signaling pathways in both virus-infected cells and immune cells such as natural killer (NK) cells, dendritic cells (DCs), and T cells. A principal mechanism by which IFN-α directly inhibits viral replication is the upregulation of key antiviral effector proteins, such as IFN-induced transmembrane proteins (IFITMs), myxovirus resistance protein 1 (MX1), and apolipoprotein B mRNA editing enzyme catalytic-like 3 (APOBEC3), which inhibit viral replication by disrupting critical steps in the viral life cycle [4,7,8]. Numerous members of this interferon-stimulated gene (ISG) family have been demonstrated to possess potent anti-HBV activity [9]. Additionally, IFN-α contributes to antiviral immunity by regulating both innate and adaptive immune responses [4].

During HBV infection, the outcome of the infection is largely determined by the response of the immune system. Most HBV infection in infants and young children will lead to chronic infection, while in adults, the likelihood of developing chronic infection is less than 5% [1]. This suggests that immune maturity and immune response control play a crucial role in determining whether the infection becomes chronic. The immune system controls the spread of HBV through the combined action of innate and adaptive immune responses [10]. The innate immune response serves as the first line of defense after infection, primarily functioning through interferons and natural killer (NK) cells. The adaptive immune response is mainly mediated by T cells, particularly CD8^+^ cytotoxic T lymphocytes (CTLs), which can directly recognize and kill hepatocytes infected with HBV. Additionally, CD4^+^ helper T cells promote B cells to produce neutralizing antibodies by secreting cytokines, thereby further inhibiting virus replication [1,11]. However, there are significant differences in immune responses between acute and chronic HBV infections [12]. In acute settings, the immune system typically recognizes and clears the virus quickly and effectively, with most patients eliminating the virus within weeks. In contrast, during chronic infections, the antiviral immune response is relatively inefficient and dysregulated, with virus-specific T cell functions being suppressed, leading to the persistence of the virus and development of chronic hepatitis B [13]. Known mechanisms of the immune evasion include T cell exhaustion, upregulation of immunoregulatory factors (such as programmed death-1, PD-1), viral strategies to escape from host immune recognition, paucity of T cell activation, and subsequent migration and liver infiltration [14,15]. Emerging evidence indicates that HBV-specific CD8^+^ T cell dysfunction often diverges from classical exhaustion [16]. Recent work by Knolle and colleagues stated that interactions between liver sinusoidal endothelial cells (LSECs) and intrahepatic T cells promote tolerance and limit immunopathology, resulting in impaired T cell receptor (TCR) kinase phosphorylation and a distinct hyporesponsive T cell state [17].

Different from general viral infections, the characteristics of the innate immune system and the interferon (IFN) system exhibit certain uniqueness in HBV infection. HBV is often considered as a stealth virus that can passively avoid triggering a strong IFN response and actively interfere with the IFN-I pathway through various mechanisms [18,19]. Typically, HBV infection does not significantly activate the type I interferon (IFN-I) signaling pathway, which could favor viral persistence [11]. Nevertheless, treatment with exogenous interferon can activate the host antiviral response, although the extent of response varies among individuals. In some patients, IFN therapy promotes viral clearance and strengthens immune control, leading to a functional cure—defined as sustained loss of HBsAg and HBV DNA without ongoing liver damage, even in the presence of a residual viral reservoir [20]. However, the overall clinical benefit of IFN-α therapy remains limited, primarily due to a suboptimal risk–benefit ratio driven by its modest response rate, substantial economic burden, and particularly, its frequent adverse effects such as flu-like symptoms and cytopenia, which arise from its pleiotropic effects as a broad-spectrum immunomodulatory cytokine on systemic tissues and organs [21]. In addition, the finite duration of IFN-α therapy and its associated adverse effects may contribute to suboptimal immune reconstitution, which can consequently lead to virological relapse or HBsAg re-elevation post-treatment, as the sustained immune pressure required to control the persistent cccDNA and integrated viral DNA reservoirs may not be fully established [13]. Therefore, strategies such as combination therapy and the development of novel, more potent interferon-based agents are being actively explored to enhance therapeutic efficacy.

## 2. Immune Modulatory Role of IFN-I in Innate Immune Cell Responses

**Figure 1:**
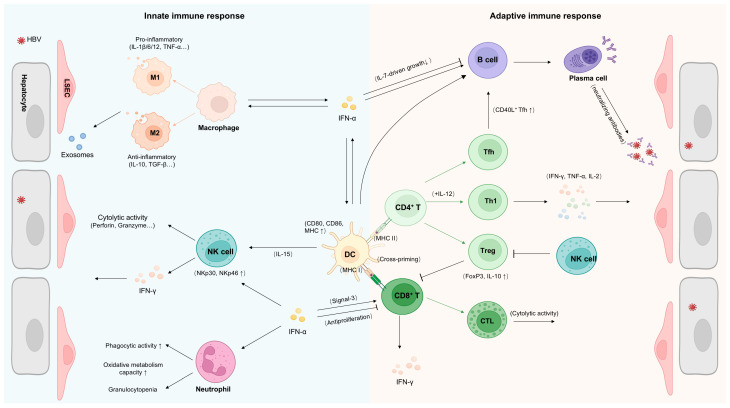
Immune mechanisms of IFN-α-mediated control of chronic HBV infection. IFN-α activates multiple immune cell populations through direct and indirect mechanisms. Specifically, IFN-α enhances the antigen-presenting capacity of DCs, promoting the activation of CD8^+^ and CD4^+^ T lymphocytes. This T cell activation consequently augments cytokine secretion and cytotoxic T lymphocyte (CTL) responses. Furthermore, IFN-α increases the proportion of plasma cells, and potentiates neutralizing antibody production against viruses. Concurrently, IFN-α amplifies NK cell responses, enhancing their cytotoxic activity. Additionally, IFN-α augments antiviral effects by inducing exosome production in non-parenchymal liver cells, including macrophages.

As essential first-line defenders, type I interferons serve as central orchestrators of innate antiviral immunity and pivotal bridges to adaptive immune activation (Figure 1). Beyond canonical antiviral effects, IFN-I critically modulate the activation, differentiation, and effector mechanisms of key innate immune cells through finely tuned signaling pathways, including natural killer cells, dendritic cells, macrophages, and neutrophils [22]. Such multifaceted regulation enables context-dependent balancing of pathogen clearance with immune homeostasis maintenance, while dysregulation may fuel immunopathology.

### 2.1. Modulatory Role of IFN-I in NK Cell Responses

The maintenance of NK cell function is largely dependent on IFN [23], and IFN-I can activate NK cells through direct or indirect pathways [24,25,26]. Its impact on NK cells is considered to be related to the induction of different signal transducers and activators of transcription (STATs). For example, IFN-I primarily impacts NK cell cytotoxicity through STAT1, while affects the capacity of secreting IFN-γ in IL-12/STAT4-dependent ways [27]. The activation of NK cells by IFN-I may also cause tissue damage, and the apoptosis of NK cells via the TNF-related apoptosis-inducing ligand (TRAIL) is considered to contribute to the immunopathological damage caused by IFN-I [28]. However, the host may also employ certain self-regulatory mechanisms to mitigate the NK cell-mediated damage [29].

IFN-α can activate NK cells when used for HBV treatment. In HBeAg-positive patients with chronic hepatitis B receiving polyethylene glycol IFN-α (PEG-IFNα) treatment, analysis of isolated peripheral blood mononuclear cells (PBMCs) showed significant improvement in NK cell responses, including enhanced proliferation, activation, and antiviral effects [30]. By comparatively monitoring the NK cell responses in the peripheral blood and liver of patients during treatment, it was found that patients with evident NK cell activation in the early stage of treatment responded better to PEG-IFNα, suggesting that the immunomodulatory effects of interferon on NK cells play a key role in therapeutic response [31]. In another work, HBeAg positive patients that received PEG-IFNα for 48 weeks followed by 9 months of sequential NUC treatment showed that compared to monotherapy, interferon treatment further enhanced NK cell proliferation after NUC therapy, upregulated the activation receptors NKp30 and NKp46, and only patients with increased IFN-γ secretion and cytotoxicity, along with downregulated TRAIL expression, were functionally cured [32]. Recent research has also found that PEG-IFNα treatment can promote the inhibitory effects of NK cells on Treg cell proliferation and differentiation [33].

### 2.2. Modulatory Role of IFN-I in Dendritic Cells

DCs, as innate immune cells with specialized antigen-presenting functions, serve as key initiators of adaptive immune responses. Among DC subsets, plasmacytoid DCs (pDCs) are the principal producers of IFN-I, whereas both pDCs and conventional DCs (cDCs) can respond to IFN-I signaling to undergo maturation and activation. The extent and nature of this response depend on the type of pathogens and the specific DC subset involved. Treatment with IFN-I in vitro can accelerate the maturation of DCs, upregulate the expression of co-stimulatory molecules on activated DCs, and enhance their ability to present antigens and activate T cells. However, the effective performance of these functions requires the involvement of other modulators, such as TNF-α [34,35]. The absence of the IFN-I pathway in DCs inhibits the maturation and activation process of DCs, demonstrating the important role of IFN-I in inducing DC maturation and activation [36]. Additionally, different DC subsets have varying responses to IFN-I [37]. However, studies in Measles virus (MV) transgenic mice and Lymphocytic choriomeningitis virus (LCMV) infection models have shown that virus-induced IFN-I may inhibit DC proliferation and maturation, which depend on the activation of STAT2 signaling [38].

Some antigen-presenting cells can utilize major histocompatibility complex class I (MHC I) molecules to present exogenous antigens to CD8^+^ T cells, a process known as cross-presentation, thereby activating naive CD8^+^ T cells. This process is referred to as cross-priming. Studies using ovalbumin (OVA) and hepatitis C virus (HCV) NS3 protein, as well as studies in the LCMV infection model, have found that IFN-I can directly activate DCs to effectively display antigens on MHC class I molecules, thereby promoting the cross-priming of CD8^+^ T cell [39,40,41]. Ellen Duong et al. demonstrated that, in contrast to DC1s which rely on cross-presentation, ISG^+^ DCs activate anti-tumor CD8^+^ T cell responses by acquiring and presenting intact tumor-derived peptide-MHC I complexes through MHC-I dressing, which is enhanced by type I interferon [42]. In addition, IFN-I can also enhance DC migration into draining lymph nodes, triggering antiviral immune responses. The migratory capacity of DCs is regulated by their responsiveness to chemokines. IFN-I can help to upregulate the expression of chemokine receptor type 7 (CCR7) in DCs and enhance their ability to respond to chemokines [43]. IFN-I also enhances the adhesion to lymphatic endothelial cells, further improving their migratory ability [44].

Early studies compared of the numbers of DCs in the peripheral blood and liver of pediatric patients with chronic hepatitis B in the IT (immune tolerance) and IA (immune activation) phases. It was found that there was a reduction in peripheral blood DC subsets in IA stage, while intrahepatic DC infiltration was observed. This infiltration was found to have a significant negative correlation with plasma HBV load and serum alanine aminotransferase (ALT) levels, suggesting that the increased infiltration of DCs in the liver may be involved in the local antiviral immune response [45]. However, impairment of DC function has been observed in HBV patients, including impaired antigen presentation ability, migration ability, and cytokine production capacity, which promotes the persistence of the virus [46]. In a study of children with chronic hepatitis B receiving IFN-α treatment, the quantity and function of pDCs and mDCs were assessed at different time points. It was found that the number and function of peripheral pDCs were restored in responders, peaking at 12 weeks after treatment. These responses were associated with viral clearance, serological conversion of e-antigen, and increased levels of peripheral mDCs and Th1 cytokines. These results suggest that pDCs may be closely related to the antiviral responses of interferon treatment [47]. Recent work by Li et al. demonstrates that intrahepatic Batf3-dependent cDC1s drive CD8^+^ T cell responses through MHC-I cross-dressing of HBsAg, rather than via cross-priming [48]. The role of IFN-α in regulating this process warrants further investigation.

### 2.3. Modulatory Role of IFN-I in Macrophages

Macrophages are also an important source of IFN-I, which can directly affect HBV by secreting IFN-I. These monocyte-derived immune cells are strategically positioned throughout tissues, including the liver (as Kupffer cells), where they constitute a first line of defense against pathogens like HBV [49]. Using a co-culture system of mouse macrophages and hepatocytes, it was found that stimulator of interferon genes (STING) agonists can induce macrophages to produce large amounts of IFN-I, thereby controlling HBV replication in hepatocytes [50]. Stimulation of macrophages with TLR agonists in vitro also effectively controls HBV through the production of IFN-I [51]. Our previous research indicated that IFN can stimulate non-parenchymal liver cells, including macrophages, to produce exosomes rich in antiviral molecules. And these exosomes can then transfer intercellularly to HBV-infected hepatocytes to exert their effects [52]. It should be noted that some studies have found that IFN-I produced by macrophages may also have an immunosuppressive effect, leading to a reduced response to IFN treatment in HCV patients [53].

Macrophages can be categorized into M1 and M2 types, with M1 primarily inducing Th1 responses that can trigger inflammation and tissue damage, while M2 primarily induces Th2 responses. By comparing M1- and M2-like macrophages in WT and IFNAR^-/-^ mice, it was found that IFN-I signaling is more inclined to affect the phenotype and function of M2-like macrophages [54]. IFN-I treatment could also enhance macrophages’ response to the Th2 cytokine IL-10 [55]. In mouse models with liver inflammation, IFN-I signaling in liver macrophages can reduce pathological damage, and may also be related to M2 activation, although further investigation has not been conducted [56,57]. The regulatory mechanism of IFN-I on macrophages remains unclear, but some studies have identified ISG15 as a key regulator in regulating macrophage functions, such as phagocytic activity and antiviral effects [58,59]. The expression of ISG15 and MxA proteins can be observed in liver macrophages of CHB patients who respond well to IFN-α therapy, while in non-responders they are primarily expressed in hepatocytes, suggesting that this may serve as a predictive indicator of the outcome of IFN-α treatment [60].

### 2.4. Modulatory Role of IFN-I in Neutrophils

Neutrophils can also participate in the production of IFN-α and can also be regulated by it [61]. Exposure to IFN-α in vivo significantly enhances the phagocytic function and infection-ability of neutrophils [62]. Chronic hepatitis C (CHC) patients show a marked increase in the oxidative metabolic capacity of neutrophils after IFN-α treatment [63]. However, during long-term treatment, the number of peripheral neutrophils decreases significantly, while the level of granulocyte colony-stimulating factor (G-CSF) in serum increases significantly. This suggests that IFN-α may stimulate the bone marrow to produce neutrophils by increasing G-CSF production to compensate for the reduction in neutrophils [64,65].

Neutrophils are crucial for viral clearance in the early stages of infection, but their excessive activation can also lead to tissue damage, necessitating a balance between antiviral effects and inflammation regulation during antiviral therapy. Previous studies using HBV transgenic mice have found that neutrophils can mediate liver damage by secreting neutrophil elastase (NE), and treatment with NE inhibitors can effectively reduce the severity of liver injury [66]. In patients with HBV-related liver failure and cirrhosis, neutrophil counts were observed to be higher than in healthy patients, and the neutrophil-to-lymphocyte ratio (NLR) was positively correlated with serum HBeAg levels [67,68]. In patients with HBV-ACLF (acute-on-chronic liver failure), although peripheral neutrophil counts were significantly increased, their phagocytic function was notably impaired, along with increased formation of neutrophil extracellular traps (NETs), which was more pronounced in patients with poor prognosis. These functional impairments indicate a significantly diminished role of neutrophils in responding to infection and inflammation [69].

## 3. Immune Modulatory Role of IFN-I in T and B Cell Responses

Beyond its impact on innate immunity, IFN enhances antiviral defense by directly or indirectly modulating adaptive immune cells, including T and B lymphocytes. Specifically, IFN-α promotes dendritic cell activation, potentiating T cell responses to viral antigens. Furthermore, it augments CD8^+^ T cell cytolytic function and B cell antibody production through the induction of key immune effector genes. Collectively, these mechanisms highlight IFN-α’s critical role in orchestrating immune-mediated viral clearance and host defense.

### 3.1. Modulatory Role of IFN-I in CD8^+^ T Cell Responses

In addition to the indirect activation of CD8^+^ T cells mediated by DCs, type I interferon (IFN-I) can also directly activate CD8^+^ T cells as a third signal, promoting their clonal expansion, secretion of IFN-γ, and cytotoxic effects [70,71,72,73,74]. The effect of IFN-I on CD8^+^ T cell proliferation and IFN-γ secretion is considered to be related to the activation of different STATs. Generally, the antiproliferative effects of IFN-I are primarily mediated by STAT1 [75,76], and IFN-I induced during viral infections can inhibit IFN-γ secretion in a STAT1-dependent manner [77]. In contrast, the induction of STAT4 promotes the production of IFN-γ [78].

Additionally, the timing of IFN-I pathway activation during viral infection can affect its regulation of CD8^+^ T cells. Studies in models such as West Nile virus (WNV), LCMV, and pAAV-HBV1.2 mice indicate that the activation of IFN-I signaling at different time points during viral infection can have varying impacts on antiviral-specific CD8^+^ T cell responses [79,80,81]. ZOU et al. demonstrated that post-infection activation of the IFN-I pathway enhances HBV-specific T cell responses and thus promotes HBV clearance. In contrast, activation of the IFN-I pathway prior to HBV replication induces hepatic immunosuppression and viral persistence [81].

The functionality of CD8^+^ T cells can also be constrained by the cytotoxic effects of NK cells, which can kill activated CD8^+^ T cells via NKG2D and perforin or through direct contact, thereby weakening the antiviral T cell responses [82,83,84]. However, IFN-I signaling on T cells may reduce NK cell attacks on CD8^+^ T cells by regulating NCR1 ligands [85], or inducing the expression of ligands for NK inhibitory receptors [86].

In early studies using a chimpanzee model of HBV infection, selective knockout of CD4^+^ or CD8^+^ T cells revealed that CD8^+^ T cells in the liver are the main cells responsible for viral clearance, mediated by both non-cytolytic and cytolytic pathways [87]. However, the effects of IFN-α on HBV-specific CD8^+^ T cells remain to be clarified. In patients undergoing PEG-IFNα treatment, assessments of CD8^+^ T cell counts and cytokine secretion ability in PBMCs at week 0, 4, 8, 12, and 24 showed that patients with a better response to PEG-IFNα exhibited stronger HBV core and env-specific CD8^+^ T cell responses [88]. In a study of sequential combination of nucleo(s/t)ide analogs (NUC) and PEG-IFNα, patients with higher levels of core-related antigens and surface antibodies at the treatment endpoint were found to elicit sustained cellular and humoral immunity, and these patients were more likely to achieve functional cure following PEG-IFNα treatment [89]. Jiang et al. employed scRNA-seq analysis of PBMCs from CHB patients, and demonstrated that PEG-IFNα treatment does not obviously induce T cell differentiation and exhaustion, while increasing the frequency of naive and central memory T cell subsets [90]. So far, studies supporting the notion that IFN-α can enhance HBV-specific CD8^+^ T cell responses were primarily conducted in mouse models. For instance, studies in HBV transgenic mice and pAVV-HBV1.2 models demonstrated improved HBV-specific CD8^+^ T cell responses following IFN-α treatment [91,92,93]. However, there is still a lack of mouse models that can fully simulate HBV infection to further elucidate the impact of IFN-α on HBV-specific CD8^+^ T cells.

Conversely, some research findings suggest that IFN-α treatment may not help to restore HBV-specific CD8^+^ T cell responses and may even have an inhibitory effect. In e antigen-positive patients treated with PEG-IFNα, the activation of overall CD8^+^ T cells was observed after the initial dosing, but failed to activate HBV-specific CD8^+^ T cell responses [94]. Similarly, no improvement in HBV-specific CD8^+^ T cell responses was observed 24 weeks after PEG-IFNα treatment [95]. Instead, there are studies that have reported a sharp decrease in CD8^+^ T cell counts following PEG-IFNα treatment [30]. Studies using IFNAR-deficient mice also suggested that a relatively large amount of antigen should be expressed in the liver to trigger HBV-specific CD8^+^ T cell expansion, and the antiviral ability of IFN-I appears to hinder HBV-specific CD8^+^ T cell responses by reducing HBV antigen expression [96]. In human liver chimeric mice infected with HBV, treatment with PEG-IFNα consistently reduced the virus load, suggesting that the anti-HBV effects of IFN-α may not require the involvement of immune cells [97]. But the immune system is present in HBV patients, and even if it does not play a key role or is suppressed during IFN-α treatment, understanding the underlying mechanisms is crucial for improving the response rates to IFN-α therapy.

### 3.2. Modulatory Role of IFN-I in CD4^+^ T Cell Responses

The differentiation of CD4^+^ T cells into follicular helper T cells (Tfh) or type 1 helper T cells (Th1) influences the balance between humoral and cellular immunity, and IFN-I signaling can regulate the differentiation process of CD4^+^ T cells [98,99], and the specific effects of IFN-I are influenced by the pathogens involved [100,101,102]. IFN-I may also promote regulatory T cell (Treg) differentiation while hindering the proliferation and differentiation of Th cells, particularly under tolerant environments [103,104]. The specific knockout of IFNAR on Tregs can impair the function of LCMV-specific CD8^+^ T cells or tumor-infiltrating lymphocytes, suggesting that IFN-I signaling within Tregs may contribute to eliciting more effective antiviral or antitumor immune responses [105,106].

Previous studies have primarily focused on the role of CD8^+^ T cells in HBV clearance. Recently, single-cell transcriptomic analyses of intrahepatic and peripheral lymphocytes from chronic hepatitis B (CHB) patients and functionally cured individuals revealed a shift toward the generation of CD4^+^ cytotoxic T cells (CD4-CTLs) in FC patients, indicating that the role of CD4^+^ T cells in the HBV clearance process should not be overlooked [107]. Treatment of PBMCs from CHB patients with IFN-α in vitro showed that IFN-α was more pronounced in the activation of CD4^+^ T cells compared to CD8^+^ T cells [108]. When clinically combined with ribavirin and PEG-IFNα, HBV-specific CD4^+^ T cell responses and Th1 cytokine secretion were detected in the liver and peripheral lymphocytes of patients only when viremia was significantly controlled and a sustained response was observed [109,110].

### 3.3. Modulatory Role of IFN-I in B Cell Responses

Similarly, IFN-I can enhance the antibody response by activating B cells directly or indirectly. Direct stimulation of B cells by IFN-I can influence B cell differentiation and virus-specific antibody responses [111,112,113]. When DCs are the only cell type that can respond to IFN-I in vivo, IFN-I can still promote class switching and antibody production, suggesting that IFN-I can also indirectly activate B cells [114]. Selective knockout of IFNAR on B or T cells weakens the effects of IFN-I on class switching and antibody responses, indicating that IFN-I receptor signaling on both B and T cells plays a crucial role in activating antibody responses [115]. In addition to promoting B cell responses, some studies have reported that IFN-I may also have a negative role on B cell responses, primarily related to mediating B cell death [116,117].

B cells during chronic HBV infection exhibit functional exhaustion and impaired antibody responses, as evidenced by reduced proliferative capacity and defective differentiation into antibody-secreting plasma cells [118,119]. In patients with CHB, HBsAg-specific B cells are predominantly characterized by a CD21^−^CD27^−^ atypical memory B cell (atMBC) phenotype. These cells demonstrate blunted maturation into plasma cells and compromised antiviral activity, which may account for the lack of detectable hepatitis B surface antibody (HBsAb) in CHB patients [120,121]. When IFN-α is used for the treatment of chronic hepatitis B (CHB), it can lead to the remodeling of B cell subsets. By comparing changes in B cell subsets in PBMCs of CHB patients before and after 24 weeks of PEG-IFNα treatment, it was found that memory B cells and effector B cells significantly increased after PEG-IFNα treatment, and surface antigen clearance was associated with changes in plasma cells. This suggests that B cell remodeling induced by PEG-IFNα treatment may play a key role in CHB therapy [122]. In another study, however, during 48 weeks of PEG-IFNα treatment in HBeAg-positive patients, changes in B cell subsets were monitored, revealing that PEG-IFNα treatment regulated the distribution of B cell subsets during therapy, but it returned to baseline after discontinuation and were unrelated to clinical outcomes [123]. By comparing patients who responded to PEG-IFNα treatment with those who did not, an increase in intrahepatic B cell infiltration was observed only in responders, and genes related to B cell activation and differentiation were upregulated in responders, suggesting that B cells may play an important role in the antiviral immune response [124].

Analysis of blood and liver samples from CHB patients treated with PEG-IFNα for 48 weeks found a significant increase in T follicular helper (Tfh) cells in responders, particularly CD40L^+^CD4^+^CXCR5^+^ Tfh cells. Blocking the CD40-CD40L signaling pathway in vitro inhibited B cell activation and IgG production, and transcriptome analysis in responders also showed upregulation of CD40L expression. This study proposed that Tfh cells expressing CD40L in CHB patients treated with PEG-IFNα can induce B cell differentiation and further improve surface antigen seroconversion rates [125]. Another study demonstrated a strong correlation between the frequency of CD40L^+^ Tfh cells and the activation status of HBsAg-specific B cells in patients receiving PEG-IFNα therapy [126]. In addition to changes in B cells after or at the end of treatment, other studies also focused on the relationship between the baseline levels of HBsAg-specific B cells and clinical outcomes. It found that 41.2% of patients with detectable HBsAg-specific B cells at baseline achieved seroconversion after PEG-IFNα treatment, while only 13.6% of patients without detectable HBsAg-specific B cells achieved seroconversion. This suggests that patients with higher baseline levels of HBsAb-specific B cells are more likely to achieve HBsAg or HBeAg seroconversion during treatment, indicating that the baseline status of B cells can be used to predict which patients are more likely to clear viral antigens and develop protective antibodies during treatment [127].

Studies also suggest that PEG-IFNα may indirectly influence T cell responses by regulating B cells. After 48 weeks of PEG-IFNα treatment in HBeAg-positive patients, a large number of CD4^+^CD38^hi^ B cells were induced, which subsequently impaired NK and T cell functions. In contrast, patients with a lower induction of CD24^+^CD38^hi^ B cells responded better to PEG-IFNα treatment, and blocking CD24 with antibodies without affecting other B cell subsets improved therapeutic efficacy [128]. In the pAAV-HBV1.2 mouse model, pIFN-α4 treatment enhanced HBV-specific CD8 T cell responses, but this was absent in B cell-deficient mice, suggesting that the promotion of HBV-specific CD8^+^ T cell responses by IFN-α depends on B cells [93].

## 4. Strategies to Enhance HBV Specific Immune Response of IFN-I

To improve the response to IFN-α therapy for chronic hepatitis B, researchers have been constantly dedicated to develop novel molecular technologies or optimize combination strategies based on IFN-α, such as optimizing delivery strategies of IFN-α molecules, identifying new IFN-α subtypes with stronger antiviral activity, and combining IFN-α with other therapies.

### 4.1. IFN-Based Cytokine Engineering

The IFN-α used clinically in the early days was an unmodified recombinant protein with a short half-life, of which the therapeutic effect was limited and was accompanied by severe side effects [129]. To address this, long-acting IFN-α conjugated with PEG molecules was then developed, which showed extended half-life and improved bioavailability through increased molecular weight. Nowadays, PEGylated-IFNα-2a (40 kD) [130] and PEGylated-IFNα-2b (12 kD) [131] are commonly used in the clinical treatment of chronic hepatitis B. Clinical trials have shown that the response rate to PEG-IFNα treatment is higher than that of traditional IFN-α (28% vs. 12%), with improved seroconversion rates (37% vs. 25%) and a lower incidence of side effects compared to traditional IFN-α (2% vs. 4%) [132]. In recent years, some studies have also involved designing other strategies to extend the half-life of IFN-α, such as conjugating it with a polypeptide containing 600 PAS residues (approximately 300 kD) [133], or constructing fusion proteins with anti-VEGFR2. These fusion proteins have prolonged half-lives, but the binding affinity of IFN-α to its receptor may be reduced, thereby resulting in decreased functional activity. To this end, researchers further utilized an IFN-α mutant with stronger binding affinity with the receptor, namely anti-VEGFR2-IFNαmut, which significantly enhanced its antitumor activity and improved the tumor microenvironment by promoting immune cell activation [134].

In addition to improving the efficacy of IFN-α by extending its half-life, researchers have also explored tumor or specific tissue-targeted IFN-α molecules to increase the local concentration and activity while reducing systemic side effects (Table 1). Generally, researchers will identify a target molecule that is specifically distributed at a particular site, and then link a ligand that specifically binds to the target receptor with IFN-α, thereby achieving targeted delivery of IFN-α. For example, conjugating a cyclic NGR peptide that specifically binds to aminopeptidase N in the tumor vasculature with IFN-α [135], or constructing a fusion protein by coupling an antibody against CD20, which is highly expressed in B-cell lymphomas, with IFN-α [136], both can increase the concentration of IFN-α at the tumor site and enhance its antitumor activity. The liver is also a popular spot for target delivery. Researchers have utilized proteins that are highly expressed in the liver, such as PD-L1 [137], or liver-specific antigens, such as the host protein asialoglycoprotein receptor (ASGPR) [138], or viral antigen peptides HBc_18-2_ and HBs_183-91_ [139]. Fusion proteins designed based on these targets have shown increased local concentrations of IFN-α in the liver, as well as enhanced antiviral effects in the liver. However, the target specificity may be insufficient, potentially leading to detectable activity in off-target tissues. Additionally, its applicability may be limited in some systemic diseases.

Some modification strategies also attempt to combine long-acting properties with targeted delivery to achieve better functional effects while reducing systemic side effects. For example, reversible lipidization has been used to modify IFN-α’s two disulfide bonds. As the disulfide bonds are gradually reduced through the body, IFN-α is slowly released into the circulation. The induced expression of 2′-5′ oligoadenylate synthetase (OAS) in the liver was higher than that of recombinant IFN-α, indicating the liver-targeting activity [140]. A fusion protein constructed by linking apolipoprotein A-I (ApoA-I) with IFN-α was primarily expressed in the liver about two hours after injection, and its half-life was also extended, similar to PEG-IFNα [141].

In addition, optimizing the delivery strategies can also increase local concentration and the biological effects of interferon to some extent. For example, using a replication-deficient Fowlpox virus to encode and express IFN-γ can enhance its local concentration at the injection spot, selectively promoting lymphocyte proliferation and inhibiting tumor progression [142]. Similarly, intratumoral injection of mRNA encoding different cytokines takes advantage of the combined benefits of different cytokines, and thus exerts efficient antitumor activity [143]. Some research teams have also utilized TIE2-expressing monocytes (TEMs), which can be specifically recruited to tumor tissues. By inserting the IFN-α gene into the hematopoietic stem cells, IFN-α can be specifically expressed in tumor tissues. However, due to the presence of a large number of monocytes in the spleen and bone marrow, IFN-α signals were also detected in these tissues [144].

### 4.2. IFN Subtype-Specific Effects

The human type I interferon (IFN-I) family includes 12 different IFN-α subtypes, as well as IFN-β and IFN-ω. Although they share similar amino acid sequences and all bind to the common receptor, IFNAR, to activate downstream signaling molecules, increasing evidence suggests that they have distinct functional effects, including immunomodulatory and antiviral activities (Table 2) [145]. Treatment of T cells and dendritic cells (DCs) in vitro with three different subtypes of human IFN-α found that the activation of the JAK/STAT pathway and the induction levels of ISGs varied among the different IFN-α subtypes. Additionally, the effects of these IFN-α subtypes were cell-specific [146]. IFN-α2 was found to enhance T cell migration, while IFN-α8 had no such effect, indicating that IFN-α subtypes differ not only quantitatively but also qualitatively [147]. However, only IFN-α2 subtype has been approved for clinical use as a therapeutic drug.

A number of studies have attempted to compare the antiviral activities of different IFN-α subtypes and aimed to identify a subtype with better efficacy than IFN-α2. The results, however, varied depending on the virus. For example, IFN-α17, -α7, and -α8 showed stronger anti-HCV activity than IFN-α2 in an HCV cell model [148]. IFN-α16, -α5, and -α4 were up to 230 times more effective in controlling IAV infection than the clinically used IFN-α2 in a human primary lung tissue infection model for IAV [149]. In a cell model infected with SARS-CoV-2, researchers categorized the 12 IFN-α subtypes into high, medium, and low groups according to their antiviral effects, with IFN-α2 classified as medium, while IFN-α5, -α4, and -α14 showed significantly stronger antiviral effects and were classified as high [150]. Using various in vitro and in vivo HIV models, including human PBMC [151], LPMC [152], T cell lines [153], as well as Hu-PBL [154] and TKO-BLT [155,156] humanized mouse models, studies have also consistently shown differences in the antiviral and immunomodulatory activity of various IFN-α subtypes. IFN-α14 has been shown to be one of the most effective subtypes against HIV. As for our lab, we have also previously compared the antiviral activity of different IFN-α subtypes against HBV in various cell models and found that IFN-α14 was the most potent subtype. Its superior anti-HBV effect over IFN-α2 was further validated in a human liver chimeric mouse model. It was revealed that IFN-α14 has stronger binding affinity to the receptor and can synergistically activate both IFN-I and IFN-II signaling pathways, thereby inducing a range of highly effective antiviral molecules. Guided by this, we further engineered the IFN-α2-EIFK variant via key amino acid substitutions within the IFNAR1-binding interface. Remarkably, IFN-α2-EIFK recapitulated IFN-α14’s potency, achieving significantly greater HBV suppression than wild-type IFN-α2 [157].

Although increasing studies have indicated that different IFN-α subtypes have varying antiviral and immunomodulatory effects, due to the interaction between IFN-α and its receptor being species-specific [158], there is currently no immunocompetent mouse model available to support the study of the different immune functions of human IFN-α subtypes. Many studies, therefore, rely on in vitro approaches [159] or use murine IFN-α to illustrate differences in the immunomodulatory functions of murine IFN-α subtypes [160,161,162]. Thus, the construction of an immunocompetent mouse model will help to identify new IFN-α subtypes with better antiviral and immunomodulatory properties against HBV, further improving the response rate of current IFN-α2 treatments [163].

**Table 2:** Differential antiviral and immunoregulatory effects of IFN-α subtypes.

IFN-α Subtypes	Antiviral Effects					Immunomodulatory Effects
	HBV [157]	HCV [148]	HIV [155]	IAV [149]	SARS-CoV-2 [150]	
IFN-α1/13	+++	+	+++	-	-	/
IFN-α2	++	++	++	+	+	Enhanced T cell motility [147]; Higher frequencies of GranzymeB and CD107a positive CD8^+^ T cells [155];
IFN-α4	+	+	+	+	++	/
IFN-α5	++	++	++	++	+++	/
IFN-α6	+++	+	+++	+	-	/
IFN-α7	++	+++	++	+	+	/
IFN-α8	++	+++	+	-	+++	Enhanced expression of IFN-γ, IL-2 and IL-4 by CD4^+^ T cells [159];
IFN-α10	++	++	+	+	-	Enhanced expression of IFN-γ, IL-2 and IL-4 by CD4^+^ T cells [159];
IFN-α14	+++	++	+++	+	+++	Increased innate immunity and higher frequencies of TRAIL^+^ NK cells [155]; Resulted in a lower naive to effector CD8^+^ T cell ratio and associated with fewer indicators of T cell dysfunction [156];
IFN-α16	+	+	+	+++	-	/
IFN-α17	++	+++	+++	+	+	/
IFN-α21	++	++	++	-	+	/

### 4.3. Combination Therapy

**Figure 2:**
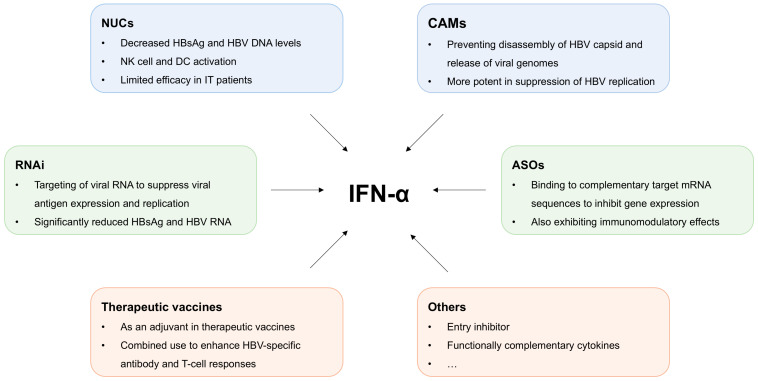
IFN-α-based combination therapies for chronic HBV infection. To enhance IFN-α therapeutic efficacy and clinical response rates in chronic hepatitis B, multiple IFN-α-based combination strategies are under investigation. These include combining IFN-α with direct-acting antivirals (DAAs) targeting distinct stages of HBV lifecycle, such as nucleos(t)ide analogs (NUCs), capsid assembly modulators (CAMs), RNAi therapeutics, and antisense oligonucleotides (ASOs), alongside immunomodulators (e.g., therapeutic vaccines and cytokines).

Given the limited efficacy of IFN-α monotherapy—owing not only to the complexity of the HBV life cycle but also to the virus-induced impairment of host immune responses—accumulating evidence supports that rational combination strategies offer superior therapeutic benefits (Figure 2) [20]. In particular, combining IFN-α with agents that target different steps of the HBV replication cycle or with immunomodulatory compounds of complementary functions has been shown to more effectively reduce viral replication, lower HBsAg burden, restore antiviral immunity, and ultimately enhance the likelihood of achieving a functional cure.

Many studies have evaluated the efficacy of combination therapy with nucleo(s/t)ide analogs (NUCs) and IFN-α in chronic hepatitis B (CHB) patients. Sequential combined use of IFN-α following NUC treatment can partially restore immune cell functions, including the proliferation and activation of NK cells and the early activation of dendritic cells (DCs) [32,164]. After 48 weeks of combination therapy with TDF and PEG-IFNα in CHB patients, HBsAg and HBV DNA levels were more substantially reduced than in the monotherapy group [165]. However, in studies involving both adult and pediatric HBeAg-positive patients in the immune-tolerant phase, the combination of entecavir and PEG-IFNα showed limited efficacy [166,167]. A recent study showed that in HBeAg-negative chronic hepatitis B patients, switching from NUCs to PEG-IFNα for 48 weeks significantly reduced virological relapse rates and resulted in higher HBsAg loss rates compared to NUC cessation alone [168].

Beyond NUCs, diverse classes of direct-acting antivirals (DAAs) have been progressively developed and deployed in combination regimens with IFN-α. The entry inhibitor Myrcludex B, a drug targeting HBV and HDV co-infections, works by preventing virus from entering into hepatocytes, and it can synergistically control HDV and HBV infections when used in combination with PEG-IFNα [169]. Capsid assembly modulators (CAMs) can stabilize the HBV capsid, preventing its disassembly and release of viral genomes, which can help to reduce viral replication and enhance the immune recognition process. Using PHH and human liver chimeric mouse models of HBV infection, it was found that combined treatment with the capsid modulator BAY41 and IFN enhances innate immune responses and synergistically exerts stronger antiviral effects [170]. NVR 3-778, also a capsid inhibitor, demonstrated stronger anti-HBV effects when used in combination with PEG-IFNα, showing more effective inhibition of HBV antigen expression and HBV replication compared to entecavir [171].

Small interfering RNA (siRNA) targeting of viral RNA is a promising strategy to suppress viral antigen expression and replication, with several candidates in clinical development [172,173]. VIR-2218 (elebsiran, BRII-835), targeting the highly conserved HBV X gene region, silences all HBV transcripts across genotypes. A study by Yuen et al. has evaluated VIR-2218 plus PEG-IFNα in NA-treated, virologically suppressed, non-cirrhotic chronic HBV patients [174]. Similarly, xalnesiran, a siRNA therapeutic collaboratively developed by Roche and Dicerna Pharmaceuticals, targets a conserved HBV genomic region to silence multiple transcripts. Recent clinical data demonstrated that in virologically suppressed, chronic HBV-infected participants receiving NAs, combination therapy with xalnesiran, and PEG-IFNα achieved superior HBsAg loss (30%) and seroconversion (23%) rates at end-of-treatment (EOT) versus comparators in this population, with rates of 23% and 20% sustained at 24 weeks post-treatment. However, treatment-emergent adverse events were common [175].

Antisense oligonucleotides (ASOs), chemically synthesized single-stranded molecules (typically 12–30 nt), inhibit gene expression by binding to complementary target mRNA sequences. Beyond directly degrading HBV RNA, ASOs also exhibit immunomodulatory effects, representing a novel therapeutic modality with significant potential for CHB. For instance, bepirovirsen, an unconjugated ASO developed collaboratively by Ionis Pharmaceuticals and GSK for CHB treatment, targets all HBV transcripts from both covalently closed circular DNA (cccDNA) and integrated HBV DNA, thereby reducing viral protein expression. Clinical research by Maria Buti et al. demonstrated that sequential bepirovirsen followed by PEG-IFNα is well-tolerated and efficacious in NA-suppressed CHB patients [176]. Another ASO, AHB-137, showed high HBsAg clearance rates, rapid responses, and a favorable safety profile in a phase IIb trial, with enhanced efficacy versus existing therapies. Phase III trials for AHB-137 are planned, alongside the exploration of combinations with interferons and PD-1 inhibitors. These findings collectively demonstrate the pivotal role of a dual strategy combining viral suppression and immune activation in enhancing functional cure rates for CHB.

Furthermore, studies have demonstrated that IFN-α can be combined with various immunomodulators as well. As a potent dendritic cell activator, IFN-α has been employed as an adjuvant in therapeutic vaccines [177]. The combination of therapeutic vaccine BRII-179 with PEG-IFNα significantly enhanced HBV-specific antibody and T-cell responses [178]. In addition, the combined use of IFN-α with functionally complementary cytokines, such as IL-15 and IL-2, can also enhance the anti-HBV immunity. Crucially, sequential IL-2 administration following PEG-IFNα therapy has been shown to improve clinical outcomes in suboptimal responders [179,180].

## 5. Concluding Remarks

Although significant progress has been made in understanding the immunomodulatory role of IFN-α in HBV infection, there are still several critical issues that require further exploration. First, despite the widespread use of IFN-α in the treatment of chronic hepatitis B, its specific immunomodulatory mechanisms, especially regarding different responses between individuals, have not been fully elucidated. Studies suggest that individual genetic factors may significantly influence their response to IFN-α, with these genetic differences likely involving certain critical genetic variations in IFN signaling pathways, thus affecting the treatment outcomes [181,182]. Therefore, further research into these genetic factors and the development of targeted therapies represent important directions for improving the efficacy of IFN treatment in the future. Additionally, some patients may develop autoantibodies against interferon or PEG during treatment with IFN-α or its long-acting formulations such as PEG-IFNα, which can reduce therapeutic efficacy and compromise antiviral responses [183,184,185]. Therefore, future research should focus more on evaluating the impact of these factors on treatment efficacy and finding strategies to prevent and overcome these challenges. Future therapies should focus more on optimizing antigen presentation and T-cell responses, particularly in patients with a high antigen load, where elevated HBV antigen may suppress HBV-specific immune responses. Through the combined use of immunomodulators, it may be possible to more effectively reduce the antigen load and enhance the antigen recognition and HBV elimination. Beyond individual immune cell regulation, IFN-I orchestrates systemic immune responses by modulating immune activity across multiple organs, including lymph nodes, spleen, bone marrow, and the liver. Recent advances in multi-omics and spatial biology technologies enable deeper insights into the spatiotemporal coordination and network interactions underlying IFN responses. These approaches may help uncover new mechanisms and therapeutic targets to enhance IFN efficacy.

Looking ahead, the development of novel, more potent IFNs, along with the integration of functional molecular targeting other immunotherapy targets or combination with antiviral drugs, could better enhance antigen presentation and T-cell activation, thereby maximizing the treatment efficacy for chronic hepatitis B. In addition, advancements in precision medicine will provide a foundation for developing personalized treatment therapy, thereby improving the response rate of IFN-α therapy while reducing the incidence of adverse effects, and improving the overall risk–benefit ratio at the population level, ultimately achieving a higher rate of functional cure.

## Figures and Tables

**Table 1:** Selective engineering approaches of IFN.

Objectives	Reagents	Approaches and Mechanisms	References
Prolonged half-life	Pegylated IFNs	Covalent attachment of an inert polyethylene glycol (PEG) polymer to the interferon molecule to produce a larger molecule with prolonged half-life.	[130,131,132]
	PAS-mIFNα	With the help of PASylation technology that adds a polypeptide comprising Proline, Alanine and Serine (PAS), mIFNα11 was fused with a 600 amino acid PAS chain to increase plasma half-life.	[133]
Tissue targeting	IFN-α2a-NGR	Coupled a cyclic NGR peptide with the C terminus of IFN-α2a, in which the NGR (Asn-Gly-Arg) peptide is a tumor-homing peptide.	[135]
	anti-CD20-mIFNα	The N-terminus of mIFNα1 or hIFNα2a was fused via a Gly_4_Ser linker to the C-terminus of the heavy chain of anti-CD20 to generate the more potent fusion proteins.	[136]
	anti-PDL1-IFNα	High level of PDL1 was observed in liver infected with HBV, and anti-PDL1-IFNα heterodimeric fusion protein could allow targeted delivery of IFNα into the liver.	[137]
	mIFNα2-ASGPR dAb fusion protein	mIFNα2 was fused to a domain antibody (dAb) specifc to a hepatocyte restricted antigen, asialoglycoprotein receptor (ASGPR), to specifically targets the liver.	[138]
	TCR-L/IFNα fusion proteins	TCR-like antibodies (TCR-L) able to selectively recognize HBV peptides were generated, and each antibody was genetically linked to two IFNα molecules to produce liver-targeted fusion proteins.	[139]
Enhanced half-life with targeting	anti-VEGFR2-IFNα (mut)	IFNα(mut) was fused with anti-VEGFR2 antibody through G4S linker to yield a novel fused antibody that showed increased half-life and significant anti-cancer activity.	[134]
	PAL-IFN	IFN-α containing two disulfide bonds was reduced and modified with a reversible lipidization agent. IFN was then slowly released from PAL-IFN into blood circulation upon reduction of the disulfide bonds in vivo (~8 h).	[140]
	ApoA-I-IFN	Systemic administration of plasmid encoding IFNα linked to ApoA-I (IA) resulted in longer half-life than IFNα and exhibited hepatic tropism.	[141]
Novel delivery platform	rF-MuIFN-γ	A replication-deficient recombinant avian (fowlpox) virus was generated to express the murine IFN-γ gene, which was able to deliver concentrated levels of the cytokine to a local tissue microenvironment.	[142]
	mRNA-encoding cytokines	Intratumoral administration of saline-formulated mRNA encoding four cytokines including IFN-α could minimize the potential for off-target effects and adverse reactions potentially associated with carrier material.	[143]
	*hTIE2*-IFN-mirT	Developed a gene transfer strategy into hematopoietic stem cells (HSCs) to target IFN-α transgene expression to tumor-infiltrating monocytes/macrophages.	[144]
